# Integration of peripheral circadian clock and energy metabolism in metabolic tissues

**DOI:** 10.1093/jmcb/mjz112

**Published:** 2019-12-21

**Authors:** Yanchen Zhang, Wenxiang Zhang, Chang Liu

**Affiliations:** State Key Laboratory of Natural Medicines and School of Life Science and Technology, China Pharmaceutical University, Nanjing 211198, China

## Abstract

**Graphical Abstract:**
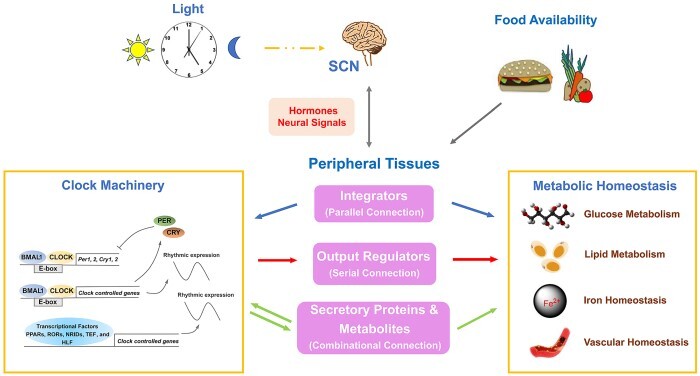

The mammalian circadian clock precisely controls various physiological processes, including energy metabolism, to help the body adapt to the diurnal light–dark cycles induced by the earth autorotation. Although the clock and metabolism are tightly coupled and interacted, the molecular mechanism through which these two pathways are integrated remains largely unknown. In the past 10 years, we have been focusing on this important scientific question and innovatively proposed three integration modes, including the ‘parallel connection’ mode orchestrated by nuclear factors, the ‘serial connection’ mode orchestrated by clock-controlled proteins, and the ‘combinational connection’ mode regulated by metabolites. Here, we summarize and discuss the molecular mechanisms of the integration between peripheral circadian clock and energy metabolism in metabolic tissues.

## Circadian clock

It is quite natural that we wake up each morning, eat our regularly timed foods, go through our daily activities, and fall asleep again when another night falls. Such a rhythm is actually controlled by the circadian clock, underlying a very exquisite mechanism through which plants and animals are able to adapt their behaviors to the 24-h change in the external environment evoked by the Earth’s rotation (Reppert and Weaver, 2002). Due to the importance of circadian clock in maintaining physiological homeostasis, the 2017 Nobel Prize in Physiology or Medicine was awarded to three scientists to honor their discoveries of molecular mechanisms controlling the circadian rhythm. Until now, it is widely recognized that circadian rhythms are remarkably conserved throughout evolution and represent a typical example of systemic biology.

The intrinsic circadian clock is mainly entrained by light–dark (LD) and feeding cycles. The mammalian master clock resides in the suprachiasmatic nucleus (SCN), a small area of the anterior hypothalamus, and drives slave oscillators distributed in various peripheral tissues through behavioral and neuroendocrine signals (Brown and Azzi, 2013). In contrast, peripheral tissues were found to contain functional clock oscillators that are self-sustained at the single-cell level. More importantly, these peripheral oscillators can be uncoupled and reset from the central pacemaker by restricted feeding, while leaving SCN rhythms unaffected (Mendoza et al., 2005). Indeed, both food availability and the temporal pattern of feeding determine the repertoire, phase, and amplitude of the circadian transcriptome in the mouse liver. Therefore, while the LD cycle resets the master clock in SCN, timed food intake is a potent synchronizer of peripheral clocks.

## Molecular architecture of the mammalian circadian clock

In mammals, the circadian clock system comprises interconnecting transcriptional–translational loops of clock genes/proteins ubiquitously expressed in various tissues and organs (Harmer et al., 2001). In the core loop, the transcriptional activators of circadian locomotor output cycles kaput (CLOCK) and brain and muscle ARNT-like 1 (BMAL1) form heterodimers, subsequently activate the expression of repressors, encoded by three Period (PER1, PER2, and PER3) and two Cryptochrome (CRY1 and CRY2) genes. Toward the end of the day, PER and CRY protein complexes translocate into the nucleus and inhibit CLOCK/BMAL1-mediated transcription. However, due to their instability, the protein and mRNA abundance of these repressors rapidly decreases below the threshold required for autorepression, thus clearing the obstacle for a new cycle. The stability of the PER and CRY proteins is regulated by specific E3 ubiquitin ligase complexes, which is important to determine the periodicity of the circadian oscillation (David and Nicolas, 2009; Gad and Ueli, 2011). In addition to these so-called ‘core circadian clock genes’, the orphan nuclear receptors of the retinoic acid receptor-related orphan receptor (ROR) and REV-ERB families are also implicated in the control of circadian clock function (Guillaumond et al., 2005).

Although the abundance may differ, the expression of clock genes is ubiquitous. Actually, the clock genes are expressed in almost all cells, just like housekeeping genes. By using circadian gene reporter technology, we now realize that essentially, every peripheral tissue or organ has the capacity to express circadian rhythms in a cell-autonomous manner. These widely distributed circadian oscillators can also function independently of the central clock located in SCN (Damiola et al., 2000). Therefore, although SCN is a master pacemaker to synchronize peripheral clocks, the body is composed of a multitude of cell-autonomous clocks that function at different hierarchical levels.

## Coupling of circadian clock and energy metabolism

The mammalian circadian clock *per se* controls diverse critical physiological activities, such as blood pressure, plasma hormone levels, sleep–wake cycles, and energy metabolism, leading to a prompt adaptation to the external changes of light and foods (Ishikawa and Shimazu, 1976). Recent studies have confirmed that in the whole mammalian genome, ~43% of the genes show rhythmic expression patterns in their target tissues and many of which encode key regulators in metabolic processes (Ray et al., 2014). For example, the promoter region of peroxisome proliferators-activated receptor α (PPARα) contains a functional E-box element, which can bind to the CLOCK/BMAL1 transcriptional complex (Canaple et al., 2006). On the other hand, it is well known that PPARα plays an important role in the fatty acid β-oxidation in peroxisome and mitochondrion (Sonoda et al., 2008). Similarly, various nuclear receptors are rhythmically expressed in those important metabolic tissues, i.e. the liver, skeletal muscle, brown adipose tissue, and white adipose tissue (Yang et al., 2006). Therefore, the circadian clock and energy metabolism are essentially coupled, and their correct coordination establishes the fundamental platform for the body health. Conversely, disruption in the normal clock function will lead to a series of metabolic disorder syndromes. A large number of epidemiological investigations have revealed that an impaired circadian clock is a risk factor for the pathogenesis of many diseases, including sleeping disorders, cardiovascular diseases, metabolic syndrome, cancers, and rheumatoid arthritis.

Given the centrality of the liver in maintaining whole-body physiological homeostasis, it is not surprising to see that almost all the hepatic functions are subjected to circadian regulation. Several high-throughput studies have been performed in mouse liver samples collected at the circadian time points, trying to reveal the regulation features of cistrome, transcriptome, proteome, and lipidome by the circadian clock (Reinke and Asher, 2016). For the glucose metabolism, glucose is phosphorylated to glucose-6-phosphate by the enzyme of G6PASE, which can either be used or stored. Of note, the circadian clock regulates all these processes, as well as controlling glucagon-induced gluconeogenesis through manipulating the duration of hepatic cAMP production. Thus, the circadian clock controls diverse mechanisms, which cooperate to generate the diurnal oscillation of glucose in the blood (Fleur et al., 2001). In addition to glucose, plasma levels of free fatty acids (FFAs), triglycerol, and cholesterol also display diurnal variations (Barnea et al., 2015). It has been well established that the circadian clock controls enzymes that are intensively involved in regulating various steps of lipid metabolism in the liver, leading to these variations in blood levels. Cellular processes, such as DNA repair, ribosome biogenesis, autophagy, and ER stress, are also under the circadian control in the liver (Sancar et al., 2010). Importantly, dysregulation of these clock-controlled pathways/processes has been proved to contribute to the pathogenesis of hepatic steatosis and other liver diseases.

## The integration modes of peripheral clock and energy metabolism

Currently, researchers have made remarkable achievements in the clock and metabolism fields, respectively. However, it remains largely unknown how these two important pathways are coupled to maintain physiological homeostasis. This is the key scientific question we have dedicated to answer in the past 10 years. According to our studies, we proposed three integration modes in which the peripheral clock and energy metabolism are coordinated.

**Figure 1:**
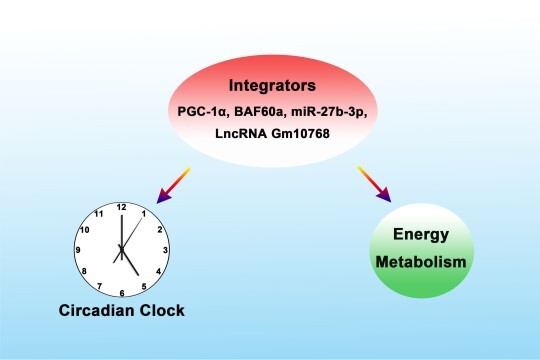
‘Parallel connection’ mode. Nuclear factors, including PGC-1α, BAF60a, and ncRNAs, serving as nodes or hubs, simultaneously regulate the peripheral clock and energy metabolism.

### The ‘parallel connection’ mode

This integration mode is mainly orchestrated by nuclear factors, as evidenced by the findings on PPARγ coactivator-1α (PGC-1α). PGC-1α was originally identified as a versatile metabolic regulator (Puigserver and Spiegelman, 2003). We found that the expression of transcriptional coactivator PGC-1α demonstrates robust diurnal oscillation in the mouse liver and skeletal muscle, and PGC-1α null mice have an abnormal physiological rhythm in their metabolic processes. At the molecular level, PGC-1α induces expression of key clock genes, such as BMAL1, CLOCK, and REV-ERBα, suggesting that PGC-1α is an upstream regulator for the clock genes. In addition, PGC-1α coordinates with ROR orphan receptors to loosen local chromatin structure, thus leading to the activation of BMAL1 transcription (Liu et al., 2007). Subsequently, we identified that BAF60a, a family member of SWI/SNF chromatin-remodeling complex and a chaperon of PGC-1α, acts as another important integrator of clock and metabolism. Specific knockdown of BAF60a in the mouse liver causes impairments in the rhythmic expression patterns of clock and metabolic genes, as well as the serum metabolite levels (Tao et al., 2011). Similar to PGC-1α, BAF60a is also able to function with RORα and induce the hepatic transcription of BMAL1 and G6PASE, typical regulators in the clock and gluconeogenesis. Interestingly, BAF60a is also an integrator in the cardiovascular system. We found that although BAF60a is rhythmically expressed both in the liver and in the layer of vascular smooth muscle cells (VSMCs) of the thoracic aorta of rodents, its rhythmicity is suppressed by high-fat diet feeding-induced hyperlipidemia. In contrast, overexpression of BAF60a in serum shock-treated VSMCs significantly restores FFA-induced attenuation of the amplitude of clock gene oscillation. 

Pathophysiologically, BAF60a inhibits VSMC proliferation and migration by blocking cell cycle re-entry and activating kinase signaling pathways. Taken together, BAF60a is a critical node integrating the circadian clock and the liver/VSMC physiological homeostasis (Chen et al., 2014a). In addition, the integrative role of non-coding RNAs (ncRNAs) has also been revealed recently. For example, we found that both miR-27 (an miRNA) and Gm10768 (a long ncRNA) exhibit rhythmic expression in the mouse metabolic tissues. These ncRNAs also respond to the nutritional signals, evidenced by an elevated expression in the liver of fasted and *ob*/*ob* obese mice. At the molecular level, the 3′UTR region of the clock gene *Bmal1* contains a potential binding site of miR-27, and miR-27 suppressed the transcriptional activity of *Bmal1* 3′UTR in the liver cells. Functionally, overexpression of miR-27 reduced the protein expression levels of BMAL1 in a dose-dependent manner and impaired the endogenous BMAL1 and gluconeogenic protein rhythmicity (Zhang et al., 2016). For Gm10768, its integrative function is achieved by the regulation of miR-214/*Atf4* axis (Cui et al., 2018).

As an overall conclusion, we believe that various nuclear factors, including PGC-1α, BAF60a, and ncRNAs, can serve as nodes or hubs, and thus simultaneously integrate the peripheral clock and energy metabolism (Figure 1). Such a pattern is similar to the parallel connection circuit in physics, so we call it the ‘parallel connection’ mode.

### The ‘series connection’ mode

**Figure 2:**
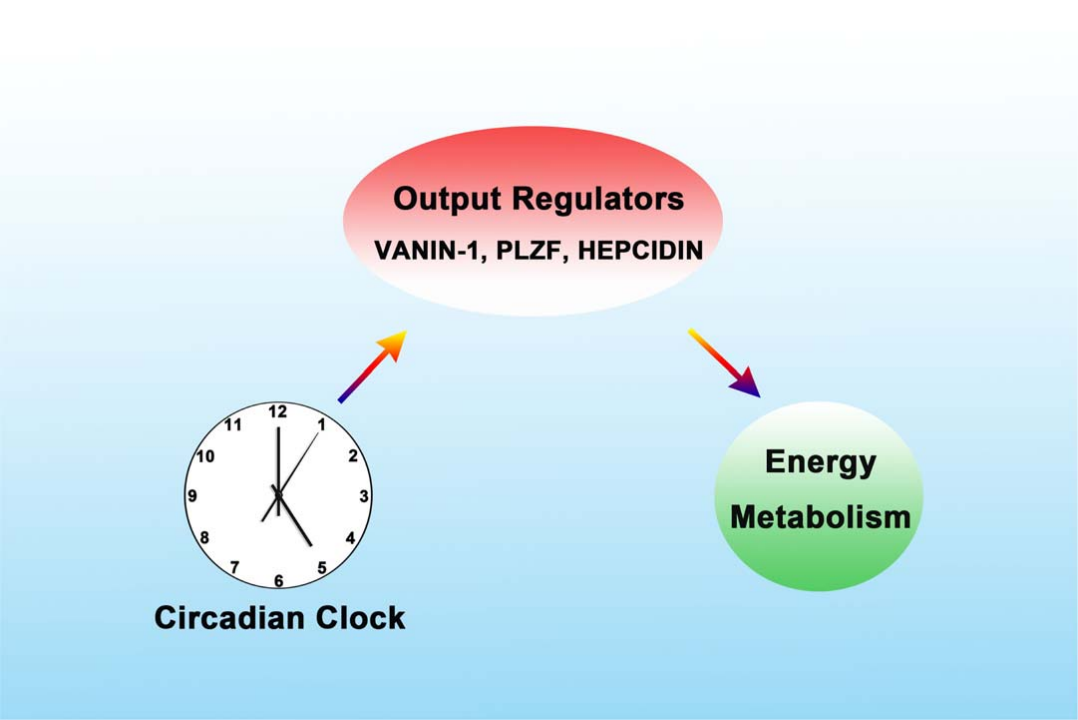
‘Series connection’ mode. CCGs including VANIN-1, PLZF, and HEPCIDIN respond to the peripheral clock signals and subsequently regulate energy metabolism as the relay mediators.

This integration mode is predominantly orchestrated by the proteins encoded by the so-called clock-controlled genes (CCGs). These proteins themselves do not act as the components of clock machinery but can respond to the clock signals and regulate metabolic processes as downstream effectors. In the mammalian genome, many genes encoding those critical enzymes involved in metabolic pathways manifest robust diurnal oscillation in their expression patterns (Ray et al., 2014). The circadian clock promotes the expression of these genes, and the accumulation of these gene products help regulate cascades of glucose and lipid metabolism (Kalsbeek et al., 2014). In our previous studies, we also tried to identify CCGs responding to PGC-1α and clock signals. Currently, at least three CCGs have been identified, i.e. VANIN-1, PLZF, and HEPCIDIN. Other researchers have confirmed that these factors play important roles in the regulation of oxidative stress and inflammation, stem cell self-renewal and differentiation, and iron metabolism, respectively. Based on these findings, we further found that these factors are rhythmically expressed in the mouse liver, and their rhythmic expression patterns can be reversed by the restricted feeding, suggesting that their expression is controlled by the peripheral clock. The proximal region of the promoters of these genes contains multiple E-box motifs, so that their transcriptional activity can be regulated by various clock genes. Finally, we found that all these factors are responsive to the coactivation of PGC-1α and nuclear factors and contribute importantly to the rhythmicity of metabolic processes such as gluconeogenesis and LPS-induced iron dysregulation. Collectively, we conclude that clock-controlled proteins, including VANIN-1, PLZF, and HEPCIDIN, respond to the peripheral clock signals and subsequently regulate energy metabolism as the relay mediators (Qian et al., 2013; Chen et al., 2014b, c; Figure 2).

### The ‘combinational connection’ mode

In this mode, secreted proteins (e.g. hormones) and metabolites respond to the upstream environmental signals and simultaneously regulate the circadian clock and energy metabolism in distal target organs/tissues through circulation (Tsang et al., 2014). Thus, this mode can be regarded as the combination of ‘parallel connection’ mode and ‘series connection’ mode. It should be noted that diurnal LD cycle resets the master clock, while timed food intake is another potent synchronizer of peripheral clocks in mammals. As the largest metabolic organ, the liver sensitively responds to the food signals and secretes hepatokines, leading to the robust regulation of metabolic and clock processes. However, it remains unknown which hepatokine mediates the food-driven resetting of the liver clock independent of the master clock. In our very recent work, we identified ANGPTL8 as a hepatokine that resets diurnal rhythms of hepatic clock and metabolic genes in mice. The resetting function of ANGPTL8 is dependent on the signal relay of the membrane receptor PIRB, phosphorylation of kinases and transcriptional factors, and consequently transient activation of the central clock gene PER1. Importantly, inhibition of ANGPTL8 signaling partially blocks food-entrained resetting of the liver clock in mice. We have thus identified ANGPTL8 as a key regulator of the liver clock in response to food (Chen et al., 2019).

**Figure 3:**
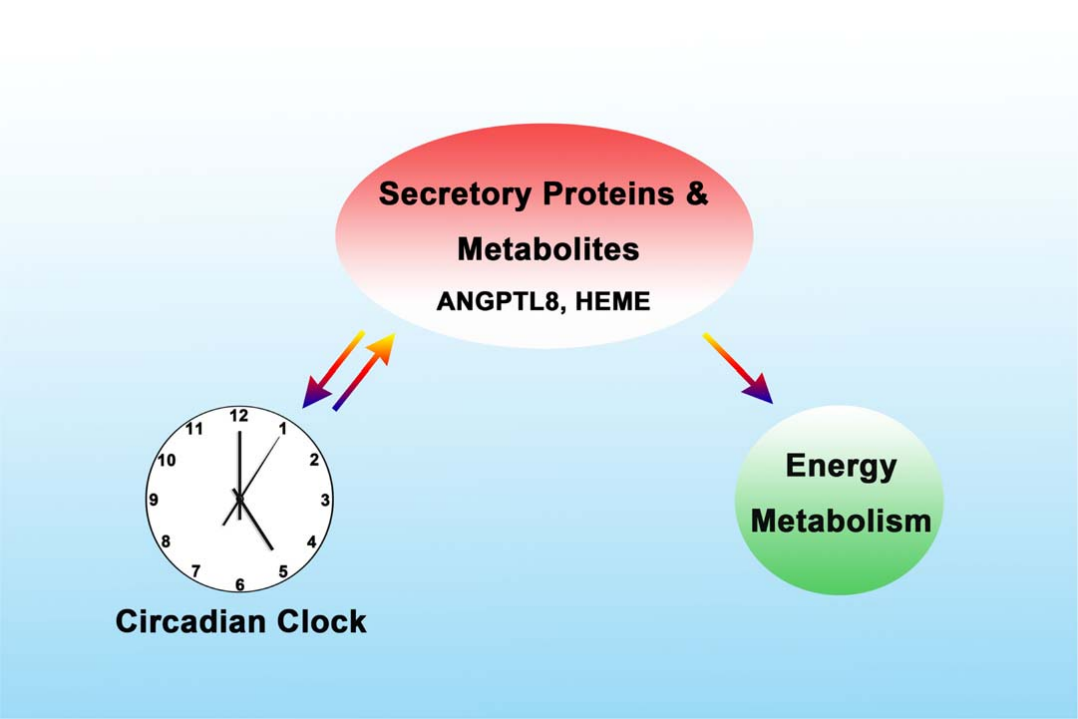
‘Combinational connection’ mode. Secreted proteins (e.g. ANGPTL8) and metabolites (e.g. HEME) respond to the upstream environmental signals and simultaneously regulate the circadian clock and energy metabolism in distal target organs/tissues through circulation.

In the study of the rhythmicity of small-molecule metabolites, the researchers found that the biosynthesis of HEME, one of the downstream metabolites of PGC-1α, is rhythmic and, in turn, directly regulates the transcriptional activity of the clock gene homolog NPAS2, leading to the entrainment of the circadian clock. HEME can also reversibly bind to the orphan nuclear receptor NR1D1, inhibiting hepatic gluconeogenic gene expression and glucose output (Yin et al., 2007). Thus, both ANGPTL8 and HEME are controlled by the circadian clock and function as the downstream rhythmic metabolites. On the other hand, these metabolites are delivered to the target tissues by circulation and manipulate the clock and metabolism in these tissues, setting up a critical feedback loop between these two pathways (Figure 3). In the future studies, we will use the metabolomics technique to screen and discover more metabolites possessing such integrative function and further synthesize specific agonists or antagonists targeting these metabolites. Our studies will aim to provide new therapeutic targets and strategies for the treatment of metabolic diseases from the view of chronobiology.

## Conclusion and remarks

To the best of our knowledge, we for the first time systemically summarized three integration modes of circadian clock and energy metabolism. In our opinion, we believe that these three integration modes are complementary and coordinate with each other to maintain the normal rhythms of metabolic processes. The nuclear factors, acting in the ‘parallel connection’ mode, are versatile regulators and hold a relatively upstream position in the integration network. Targeting these factors can simultaneously regulate clock and metabolism, achieving the effect of ‘killing two birds with one stone’. Therefore, such a regulation is multi-functional but may lose specificity and safety, since these factors are ubiquitously distributed. In contrast, the CCGs acting in the ‘series connection’ mode can remedy such shortcoming and finely regulate metabolic processes in response to clock signals in a tissue-specific manner. Finally, metabolites acting in the ‘combinational connection’ mode offer a distant regulation and establish a complicate crosstalk among different metabolic tissues.

As a summary, the systemic physiological homeostasis is not achieved by a single organ operation but rather the result of different inputs and outputs of clock signals generated from various tissues. Hence, a better understanding of the hierarchical crosstalk between clock and metabolism in multiple organs is required for studying the global control of metabolic pathways by the circadian system. In addition, a circadian viewpoint on metabolic control is valuable for researchers in the metabolism field, because new information are likely revealed when re-examining the ‘old’ metabolic pathways in a time-of-day manner.

*[We thank Dr Siyu Chen for his constructive discussion and help during the preparation of this article. This work was financially supported by grants from the National Natural Science Foundation of China (31771298 and 81800512), the Natural Science Foundation of Jiangsu Province of China (BK20180577), the Double First-Class University Project (CPU2018GY17 and CPU2018GY18), the Project of State Key Laboratory of Natural Medicines, China Pharmaceutical University (SKLNMZZRC201803), and the Open Fund of State Key Laboratory of Pharmaceutical Biotechnology, Nanjing University, China (KF-GN-201901).]*

## References

[ref1] Barnea, M., Chapnik, N., Genzer, Y., et al. (2015). The circadian clock machinery controls adiponectin expression. Mol. Cell. Endocrinol. 399, 284–287. 25448847 10.1016/j.mce.2014.10.018

[ref2] Brown, S.A., and Azzi, A. (2013). Peripheral circadian oscillators in mammals. Handb. Exp. Pharmacol. 18, 45. 10.1007/978-3-642-25950-0_3 23604475

[ref3] Canaple, L., Rambaud, J., Dkhissibenyahya, O., et al. (2006). Reciprocal regulation of brain and muscle Arnt-like protein 1 and peroxisome proliferator-activated receptor α defines a novel positive feedback loop in the rodent liver circadian clock. Mol. Endocrinol. 20, 1715–1727. 16556735 10.1210/me.2006-0052

[ref4] Chen, S., Ding, Y., Zhang, Z., et al. (2014a). Hyperlipidaemia impairs the circadian clock and physiological homeostasis of vascular smooth muscle cells via the suppression of Smarcd1. J. Pathol. 233, 159–169. 24615205 10.1002/path.4338

[ref5] Chen, S., Feng, M., Zhang, S., et al. (2019). Angptl8 mediates food-driven resetting of hepatic circadian clock in mice. Nat. Commun. 10, 3518. 31388006 10.1038/s41467-019-11513-1 PMC6684615

[ref6] Chen, S., Qian, J., Shi, X., et al. (2014b). Control of hepatic gluconeogenesis by the promyelocytic leukemia zinc finger protein. Mol. Endocrinol. 28, 1987–1998. 25333514 10.1210/me.2014-1164 PMC5414782

[ref7] Chen, S., Zhang, W., Tang, C., et al. (2014c). Vanin-1 is a key activator for hepatic gluconeogenesis. Diabetes 63, 2073–2085. 24550194 10.2337/db13-0788

[ref8] Cui, X., Tan, J., Shi, Y., et al. (2018). The long non-coding RNA Gm10768 activates hepatic gluconeogenesis by sequestering microRNA-214 in mice. J. Biol. Chem. 293, 4097–4109. 29363576 10.1074/jbc.M117.812818 PMC5857995

[ref9] Damiola, F., Minh, N.L., Preitner, N., et al. (2000). Restricted feeding uncouples circadian oscillators in peripheral tissues from the central pacemaker in the suprachiasmatic nucleus. Genes Dev. 14, 2950–2961. 11114885 10.1101/gad.183500 PMC317100

[ref10] David, D., and Nicolas, C. (2009). The crosstalk between physiology and circadian clock proteins. Chronobiol. Int. 26, 1479–1513. 20030537 10.3109/07420520903497575

[ref11] Fleur, S.E.L., Kalsbeek, A., Wortel, J., et al. (2001). A daily rhythm in glucose tolerance. Diabetes 50, 1237. 11375322 10.2337/diabetes.50.6.1237

[ref12] Gad, A., and Ueli, S. (2011). Crosstalk between components of circadian and metabolic cycles in mammals. Cell Metab. 13, 125–137. 21284980 10.1016/j.cmet.2011.01.006

[ref13] Guillaumond, F., Dardente, H., Giguere, V., et al. (2005). Differential control of Bmal1 circadian transcription by REV-ERB and ROR nuclear receptors. J. Biol. Rhythm. 20, 391. 10.1177/0748730405277232 16267379

[ref14] Harmer, S.L., Panda, S., and Kay, S.A. (2001). Molecular bases of circadian rhythms. Annu. Rev. Cell Dev. Biol. 17, 215. 11687489 10.1146/annurev.cellbio.17.1.215

[ref15] Ishikawa, K., and Shimazu, T. (1976). Daily rhythms of glycogen synthetase and phosphorylase activities in rat liver: influences of food and light. Life Sci. 19, 1873–1878. 826747 10.1016/0024-3205(76)90119-3

[ref16] Kalsbeek, A., Fleur, S.L., and Fliers, E. (2014). Circadian control of glucose metabolism. Mol. Metab. 3, 372–383. 24944897 10.1016/j.molmet.2014.03.002 PMC4060304

[ref17] Liu, C., Li, S., Liu, T., et al. (2007). Transcriptional coactivator PGC-1α integrates the mammalian clock and energy metabolism. Nature 447, 477–481. 17476214 10.1038/nature05767

[ref18] Mendoza, J., Graff, C., Dardente, H., et al. (2005). Feeding cues alter clock gene oscillations and photic responses in the suprachiasmatic nuclei of mice exposed to a light/dark cycle. J. Neurosci. 25, 1514–1522. 15703405 10.1523/JNEUROSCI.4397-04.2005 PMC6725981

[ref19] Puigserver, P., and Spiegelman, B.M. (2003). Peroxisome proliferator-activated receptor-gamma coactivator 1α (PGC-1α): transcriptional coactivator and metabolic regulator. Endocr. Rev. 24, 78. 12588810 10.1210/er.2002-0012

[ref20] Qian, J., Chen, S., Huang, Y., et al. (2013). PGC-1α regulates hepatic hepcidin expression and iron homeostasis in response to inflammation. Mol. Endocrinol. 27, 683–692. 23438894 10.1210/me.2012-1345 PMC5416804

[ref21] Ray, Z., Lahens, N.F., Ballance, H.I., et al. (2014). A circadian gene expression atlas in mammals: implications for biology and medicine. Proc. Natl Acad. Sci. USA 111, 16219–16224. 25349387 10.1073/pnas.1408886111 PMC4234565

[ref22] Reinke, H., and Asher, G. (2016). Circadian clock control of liver metabolic functions. Gastroenterology 150, 574–580. 26657326 10.1053/j.gastro.2015.11.043

[ref23] Reppert, S.M., and Weaver, D.R. (2002). Coordination of circadian timing in mammals. Nature 418, 935–941. 12198538 10.1038/nature00965

[ref24] Sancar, A., Lindsey-Boltz, L.A., Kang, T.H., et al. (2010). Circadian clock control of the cellular response to DNA damage. FEBS Lett. 584, 2618–2625. 20227409 10.1016/j.febslet.2010.03.017 PMC2878924

[ref25] Sonoda, J., Pei, L., and Evans, R.M. (2008). Nuclear receptors: decoding metabolic disease. FEBS Lett. 582, 2–9. 18023286 10.1016/j.febslet.2007.11.016 PMC2254310

[ref26] Tao, W., Chen, S., Shi, G., et al. (2011). SWItch/sucrose nonfermentable (SWI/SNF) complex subunit BAF60a integrates hepatic circadian clock and energy metabolism. Hepatology 54, 1410–1420. 21725993 10.1002/hep.24514

[ref27] Tsang, A.H., Barclay, J.L., and Henrik, O. (2014). Interactions between endocrine and circadian systems. J. Mol. Endocrinol. 52, 1–16. 23997239 10.1530/JME-13-0118

[ref28] Yang, X., Downes, M., Yu, R.T., et al. (2006). Nuclear receptor expression links the circadian clock to metabolism. Cell 126, 801–810. 16923398 10.1016/j.cell.2006.06.050

[ref29] Yin, L., Wu, N., Curtin, J.C., et al. (2007). Rev-erbα, a heme sensor that coordinates metabolic and circadian pathways. Science 318, 1786–1789. 18006707 10.1126/science.1150179

[ref30] Zhang, W., Wang, P., Chen, S., et al. (2016). Rhythmic expression of miR-27b-3p targets the clock gene Bmal1 at the posttranscriptional level in the mouse liver. FASEB J. 30, 2151–2160. 26919869 10.1096/fj.201500120

